# Resveratrol and Its Role in the Management of B-Cell Malignancies—A Recent Update

**DOI:** 10.3390/biomedicines11010221

**Published:** 2023-01-15

**Authors:** Dhruv Sanjay Gupta, Vaishnavi Gadi, Ginpreet Kaur, Meena Chintamaneni, Hardeep Singh Tuli, Seema Ramniwas, Gautam Sethi

**Affiliations:** 1 Department of Pharmacology, Shobhaben Pratapbhai Patel School of Pharmacy and Technology Management, SVKM’S NMIMS, Vile Parle-West, Mumbai 400056, India; 2 Department of Biotechnology, Maharishi Markandeshwar Engineering College, Maharishi Markandeshwar (Deemed to Be University), Mullana, Ambala 133207, India; 3 University Centre for Research and Development, University Institute of Pharmaceutical Sciences, Chandigarh University, Gharuan, Mohali 140413, India; 4 Department of Pharmacology, Yong Loo Lin School of Medicine, National University of Singapore, Singapore 117600, Singapore

**Keywords:** inflammation, B cell malignancy, resveratrol, phytotherapy, chemoprevention

## Abstract

The growing incidence of B cell malignancies globally has prompted research on the pharmacological properties of phytoconstituents in cancer management. Resveratrol, a polyphenolic stilbenoid widely found in nature, has been explored for its anti-inflammatory and antioxidant properties, and promising results from different pre-clinical studies have indicated its potential for management of B cell malignancies. However, these claims must be substantiated by a greater number of clinical trials in diverse populations, in order to establish its safety and efficacy profile. In addition to this, there is a need to explore nanodelivery of this agent, owing to its poor solubility, which in turn may impact its bioavailability. This review aims to offer an overview of the occurrence and pathogenesis of B cell malignancies with a special focus on the inflammatory pathways involved, the mechanism of actions of resveratrol and its pharmacokinetic profile, results from pre-clinical and clinical studies, as well as an overview of the marketed formulations. The authors have also presented their opinion on the various challenges associated with the clinical development of resveratrol and future perspectives regarding therapeutic applications of this agent.

## 1. Introduction

In the decades following the identification of B cells, there has been significant progress in understanding their function and distribution in biological systems, as well as the role played in pathogenesis. Three main B cell populations, namely B-1 (chiefly originating in the fetal liver, further classified as B-1a and B-1b subtypes), B-2 (produced in the bone marrow, further classified as follicular and marginal zone cells) and regulatory B cells (responsible for regulating immune responses, by countering the action of key inflammatory markers, such as interleukins) [1].

One of the integral functions of the B cells is the management of humoral immunity, a process that is delicately regulated by the transitioning of these cells from their resting stage to an active stage, followed by proliferation and the triggering of antibody production. These immune responses are tightly controlled by the balance maintained between various triggers, such as external stimuli, metabolic responses and signaling at a cellular level. Any disruptions in this homeostatic mechanism have been linked with the onset of malignancies. The key factor favoring oncogenesis is the dysregulation of important regulators associated with cellular proliferation, namely myelocytomatosis virus oncogene cellular homolog (Myc) and BCL2/TP53 [2]. Mutations in these genes have been associated with the onset and progression of malignancies. Altered B cell expression has been attributed to a majority of cancers of the immune system, with typical precursor B cell malignance (B-ALL/LBL) and malignancies of mature B cells (Hodgkin lymphoma and B cell non-Hodgkin lymphoma) gaining clinical significance [1].

Conventional treatments for B-cell malignancies, such as chemotherapeutic strategies, surgical intervention and radiotherapy pose a number of drawbacks, such as tumor-cell resistance, possibilities of damage to healthy cells, toxicity at a systemic level and potential long-term damage. In order to address these challenges, phytotherapy has been proposed as a promising line of treatment [3]. The scope of this strategy has been extended to the use of nanoformulations in cancer management, to facilitate targeted delivery and enhance therapeutic outcomes. The usage of nanoformulations of phytopharmaceuticals has been seen to improve solubility and in turn, bioavailability, as well as an enhancement in therapeutic potential, as opposed to the conventional delivery of these agents [4,5,6,7]. Another noteworthy advancement has been the application of immunotherapy. Chimeric antigen receptor (CAR)-T cell therapy has been utilized in the management of aggressive B-cell lymphomas. It has been widely studied in the management of cases of relapse, with rapid regulatory approval being obtained for second generation anti-CD19 drugs [8]. Since the approval of the first therapy for B-cell malignancies in 2017, there have been several advancements and clinical trials to determine the efficacy of this line of therapy. However, certain challenges associated with delivery and toxicity must be addressed, before leading to their wider application [9].

Resveratrol, a naturally occurring polyphenolic stilbenoid, has been steadily gaining attention in the management of cancer, owing to its abundance in nature as well as satisfactory tolerance in patients, in addition to marked health benefits following administration [10,11]. Studies have highlighted the antioxidant, anti-inflammatory and anti-carcinogenic activities of this molecule, mainly linked with the suppression of oxidative stress. In addition to this, it has been seen to mediate autophagy and exert several protective effects on a systemic level, by regulating key homeostatic mechanisms in the body [12,13]. As the search for biological targets for disease alleviation continues to grow, using computer-aided drug design and other in silico models, the interaction of resveratrol with various biological targets, such as tubulin and protein kinase C alpha (PKCα) has been highlighted. Owing to the presence of hydroxyl groups in its structure, it has been observed to undertake hydrogen bond formation as well as hydrophobic interactions at the binding site, enhancing its role in the inhibition of a variety of targets involved in pathogenic mechanisms [14].

As the therapeutic potential of resveratrol continues to be explored, there is a need to undertake a greater number of clinical trials, to cultivate a sound understanding of the therapeutic efficacy and safety profile of this agent. A greater number of studies would, in turn, encourage the development of novel drug delivery systems such as nanoparticles and nanoemulsions, to combat the drawbacks posed by conventional drug delivery systems. This would enhance therapeutic outcomes, improve the overall quality of life of patients, increase remission time with specific emphasis on cancer, and expedite the achievement of the desired health outcomes.

## 2. Inflammatory Markers and Key Pathways Involved in B Cell Malignancies

This section offers a discussion of the major inflammatory markers and pathways associated with the progression of B-cell malignancies, as well as the potential targets that may be explored to arrest cellular proliferation and aid cancer management.

Acute impairment of the immune system which is widely observed in the case of HIV infections or organ transplants serves as a high-risk factor of developing non-Hodgkin lymphoma [15]. Among HIV patients, the dysregulated increase in Epstein–Barr virus (EBV)-transformed B-cells due to damaged T-cells has been reported to induce such a B-cell malignancy [16]. Another mechanism promoting the propagation of this disease is the chronic B-cell activation leading to downstream processes, enhancing mutations as well as translocations [17].

Recently, studies have established a link between non-Hodgkin lymphoma development and changes in the levels of immunological markers including chemokines, cytokines, interleukins, as well as some soluble receptors [18]. A study conducted by Solomon et al. concluded that increased levels of TNF-α CXCL13, sCD23, sCD27, and sCD30 are directly linked with a high risk of developing NHL, indicating the potential use of these biomarkers in disease progression and prediction [19]. Newer therapeutic agents continue to be studied, in order to determine their activity in targeting the inflammatory cascades associated with the release of these markers, as well as effects exerted in arresting them.

The chemokine receptor CXC chemokine receptor 4 (CXCR4) and its ligand, CXC motif ligand 12 (CXCL12) are essential mediators of the interlinkage between tumorigenic cells of acute myeloid leukemia and the bone marrow microenvironment [20]. The CXCL12/CXCR4 signalling pathway is also one of the key pathways involved in promoting the progression, migration and angiogenesis associated with chronic lymphocytic leukemia [21]. In a study conducted by Cao et al., the expression of these receptors was correlated with disease progression, and a direct linkage was observed. This indicates the importance of monitoring the expression of these receptors, as well as considering them as targets for disease management [22].

The Wnt/β-catenin signaling pathway is a protected signaling axis that plays a role in various biological activities including the differentiation as well as adult tissue homeostasis and its dysregulation has been associated with acute lymphoblastic leukemia. Epigenetic alterations or changes in the balance of the TCF/LEF complex could lead to the hyperactivity of the Wnt/β-catenin pathway [23]. As this pathway has been primarily linked with the proliferation of cells and disease progression, exploration of inhibitors of this pathway may be a promising strategy in the reduction in disease burden and improvement in therapeutic outcomes [23].

Additionally, the Notch signaling pathway as well as the adhesion molecules are found to be the main pathways affected by the therapeutic agents to target the leukemic niche in the bone marrow microenvironment [24,25]. Recent research has linked the activation of the Notch pathway with the low-density-lipoprotein-receptor-related protein 1 (LRP1), and its inhibition may be explored as a potential target for leukemia management [26].

## 3. Molecular Mechanisms of Resveratrol in the Management of B-Cell Malignancies

### 3.1. General Mechanism in the Management of Malignancies

The potential of resveratrol in the management of neoplasms and autoimmune diseases have been explored, and great interest has been generated owing to promising pre-clinical findings. With a specific focus on B-cells, resveratrol has been observed to boost cellular autophagy by regulating the levels of key proteins involved, such as ATG5/LC3-II (upregulated by resveratrol) and p62, which showed a marked decrease following treatment. This, in turn, was linked with lowered levels of basal or human soluble BAFF (hsBAFF), thereby selectively targeting malignant cells and promoting the survival of normal cells. In addition to this, other mechanisms of autophagy have been proposed, such as inhibition of mTOR with rapamycin or possible knockdown, directly influencing cellular viability [27].

Resveratrol has also been observed to trigger apoptosis via the p53/AMP-activated protein kinase pathway, thereby inhibiting mTOR phosphorylation, in addition to downregulating the level of anti-apoptosis genes, such as Bcl-2. This, in turn, was found to inhibit the migration and proliferation of cancer cells [28]. In supplementation to the apoptotic mechanism of resveratrol, it has been observed to mediate the apoptosis of LNCaP and LNCaP-B cells even at low concentrations, by regulation of protein kinase phosphorylation, as well as the inhibition of mRNA expression. This mechanism may be prospectively harnessed for the management of prostate cancer, as well as a variety of other cancers relying on the targets highlighted [29].

With respect to the antioxidant properties of this phytoconstituent, it has been reported to control the expression of cyclin-dependent kinase inhibitors such as p21 and p27, Bcl-2 as well as cyclin B. The reduction in the expression of these proteins thereby reduced the accumulation of reactive oxygen species and endoplasmic reticulum stress [30]. Resveratrol administration, in conjunction with arsenic trioxide, has yielded benefits in neuroblastoma management, by regulating cellular viability and apoptosis, brought about by reduced levels of caspase-3 and caspase-9, as well as Bcl-2. This, in turn, was observed to markedly reduce oxidative stress [31]. Resveratrol has also been explored for its role in hepatocellular carcinoma management, and the main effects were observed to be downregulation of Bcl-2, caspase-3 and caspase-7, along with increased phosphorylation of phosphoinositide 3-kinase (PI3K) and serine/threonine-protein kinase (AKT) as well as sirtuin-1 (SIRT-1) activation, which in turn reduces cellular proliferation and migration [32].

The potential of different phytoconstituents in conjunction with conventional chemotherapeutic agents have also been explored, mainly to reduce tolerance to synthetic agents [33,34]. Resveratrol has been utilized to reduce sensitivity of breast cancer cells to Adriamycin, by regulation of microRNA (miRNA) levels, by modulation of miR-122-5p levels, responsible for the regulation of cellular apoptosis. This, in turn, lowers the expression of antiapoptotic proteins, thereby enhancing therapeutic outcomes [35].

In addition to the chemotherapeutic agents, the effects of resveratrol in combination with radiotherapy have also been assessed [36]. It was observed to reduce the viability of pituitary adenoma cells, alongside the induction of apoptosis and cellular necrosis. This may be a beneficial therapeutic outcome, as it may enable the usage of radiation of lowered intensity in conjunction with natural agents [37].

### 3.2. Molecular Mechanisms of Resveratrol with a Special Focus on B-Cell Malignancies

With special reference to B cell malignancies, the pharmacological effects of resveratrol have been highlighted in detail below.

In acute myeloid leukaemia, resveratrol can induce apoptosis and inhibit proliferation by downregulating the release of VGEF and by cell cycle arrest in the S-phase in U937 cell line [38]. Resveratrol has also been shown to inhibit tumor growth by down-regulating Bcl-2 as well as by decreasing DNA synthesis in HL60 cell line [39].

Further, in case of chronic myeloid leukaemia, resveratrol shows anti-proliferative as well as apoptosis inducing activity by inhibiting PI3K phosphorylation, reducing cyclinD1 and upregulating Caspase-3 in K562 cell line [40]. In a study utilizing the same cell line, resveratrol was also reported to induce autophagy through AMPK activation as well as JNK mediated p62/SQSTM1 expression [41].

Resveratrol induces apoptosis in acute lymphoblastic leukaemia (ALL) cells by involving a mitochondria/Caspase 9-specific pathway to activate Caspase cascade, working independently of the CD95-signaling [42]. Additionally, it promotes cell cycle arrest in S-phase and induces apoptosis through the p16/INK4A pathway in CEM-C7H2 cell line in the case of ALL types of leukaemia [43].

Lastly, in the case of chronic lymphocytic leukaemia, resveratrol was seen to inhibit proliferation and induce apoptosis by enhancing the reactivity of Caspase-3 and by cell cycle arrest in 232B4 cell line [44]. Additionally, in a study conducted using WSU-CLL cell line, resveratrol was found to induce apoptosis, and lead to the cell cycle arrest in G2/M phase by increasing caspase reactivity and by causing the accumulation of cyclins A and B [45].

With respect to cancer management, the potential for usage of resveratrol as a prophylactic agent have been explored, in addition to management strategies post disease onset. It is readily available from a number of natural sources, such as berries, grapes and other vine plants [46]. Owing to a good tolerance of the compound, its administration has been linked with the prophylaxis of several diseases with an underlying inflammatory basis, such as neurodegeneration, metabolic disorders and cancer [10]. Therefore, this agent has been explored for both the prevention as well as management of a variety of cancers.

**Figure 1:**
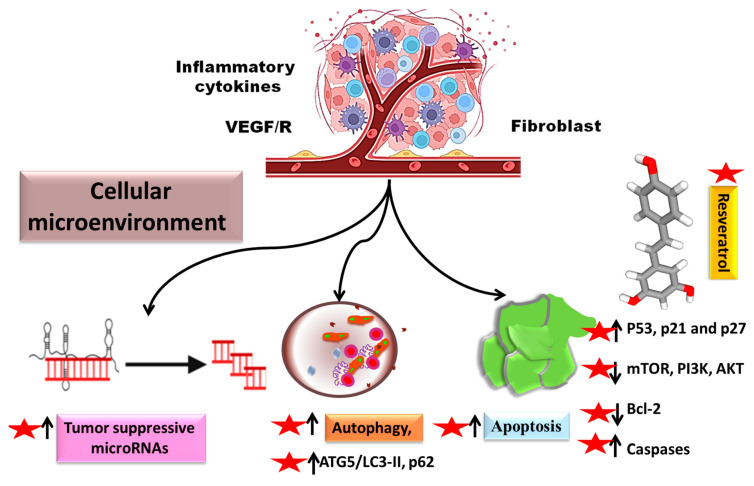
Mechanisms of action of resveratrol in the management of B-cell malignancies.

Figure 1 shows an overview of the various molecular mechanisms of resveratrol involved in the management of B-cell malignancies

## 4. Pre-Clinical Studies

A number of pre-clinical studies have elucidated the role of resveratrol in the management of B-cell malignancies. The results from recently conducted studies have been summarized in Table 1, in order to highlight the rationale of its usage and explore the possibility of further translation to clinical trials. The process of translation to clinical trials is still in its nascent stage, and the results from these studies may be used to accelerate the process. Table 1 offers a comprehensive insight into the recent pre-clinical studies of resveratrol in the management of B-cell malignancies.

## 5. Pharmacokinetic Studies of Resveratrol

Various clinical trials have been carried out with the objective of assessing the pharmacokinetic profile of resveratrol. In a study carried out by Goldberg et al., it was observed that circulating concentrations of resveratrol in the blood caused by the consumption of dietary sources such as grapes or red wine is not adequate to elicit the desired pharmacological effects, based on that required for anti-cancer activity in cultured cells in vitro [55].

A latest phase I dose escalation study assessed the safety as well as the pharmacokinetic profile of resveratrol which was administered as a single dose. The inference was that even though the bioavailability of resveratrol was not high, accumulation of resveratrol in epithelial cells and probable active resveratrol metabolites could still exhibit anti-cancer effects [56].

A study involving colorectal cancer patients concluded that even though it is practicable to achieve high levels of resveratrol in the colon, the pharmacokinetic profile of the molecule in other body tissues may be required to be closely associated with the plasma concentration [57].

Resveratrol’s activity in the internal tissues is highly dependent on the inherent action of the metabolites present and their capability to regenerate the parent molecule [58]. Recent data on the anti-cancer activity of resveratrol metabolites shows that the 3- and 4 -O-sulphates are involved in numerous mechanisms supporting its anticancer activity [59].

The half-life of resveratrol and its active metabolites has been recorded as 9.2 0.6 h when administered orally (25 mg) and 11.4 1.1 h when administered as i.v. (0.2 mg) in human subjects. The AUC was found to be 6240 680 g h/L after oral administration and 66.6 11.8 g h/L after i.v. administration [56].

It is seen that resveratrol’s stability aspects includes redox reactions as well as biotransformation that have effects on its antioxidant activity, cellular targets and chemopreventive property [60]. Trans-resveratrol is highly prone to photostability issues due to photooxidation but researchers have been addressing this by their ongoing research on resveratrol prodrugs [61].

Aliphatic derivatives of resveratrol and solid lipidic nanoparticles such as zinc/calcium-pectinate-optimized beads are have been prepared to enhance its stability, bioavailability, and drug delivery to its specific site of action [62].

Nanoparticles have advantages over conventional chemotherapeutic drugs, such as specific delivery of drugs to the tumor sites, low systemic exposures, and minimized toxicity to healthy cells. For these reasons, resveratrol loaded nanoparticles are being developed to enhance their anti-cancer, anti-oxidant and anti-inflammatory properties, thereby proving to be effective chemopreventive agents both in vitro and in vivo. Nanotechnology has successfully enhanced the oral bioavailability, stability, solubility and markedly improved the anti-cancer activity of resveratrol [63].

Several O/W nano emulsion-based drug delivery systems have been developed to enhance the bioavailability of encapsulated resveratrol for convenient oral administration. Additionally, these developed systems have also established the ability of nano emulsions in sustained release of resveratrol from dialysis bags in contrast to the unencapsulated resveratrol [64].

Furthermore, various isomers of resveratrol are being tested in clinical trials to maximize the efficacy of the compound. The micro- particulate system has also been able to efficiently deliver liquid as well as solid microparticles of resveratrol to enhance its stability. Resveratrol nano sponge formulations have innovative nano drug delivery systems, making it feasible to use the agent for its antioxidant potential to a higher extent [65].

With reference to the analogues of resveratrol to enhance its anti-cancer efficacy, in silico studies involving structural modification have revealed certain analogues that may be synthesized, in order to improve selectivity to receptors. In a study conducted by Kobylka et al., modifications at positions 3,4,5 as well as 3′,4′ and 5′ were assessed, indicating improved pharmacological actions of the visualized stilbene derivatives [66]. In a recently conducted study, several derivatives of resveratrol were explored, including methoxylated derivatives (such as pterostilbene, as well as hydroxylated derivatives (such as dihydroxystilbene).

Other approaches to the synthesis of analogues include replacement of the C=C bonds between the aromatic rings with C=N or N=N bonds, thereby improving therapeutic potential through structure modification [67], as well as π-bond extended derivatives, showing improved antioxidant and anti-inflammatory activities as compared to unmodified resveratrol [68].

Key advantages of these derivatives included improved stability, possibilities for expansion of the spectrum of biological activities, as well as the exploration of therapy in combination with other agents [69]. In addition to the ever-widening scope for anti-cancer applications, the benefits of the analogues synthesized have also been explored for the management of neurodegenerative diseases (chiefly by targeting monoamine oxidases) [70], a variety of metabolic disorders (owing to its activity as a partial agonist of the PPAR-γ receptor) [71], as well as antileishmanial effects (chiefly by triggering cell cycle arrest and increasing free radical accumulation [72]. This highlights the need to undertake a greater number of studies to uncover the properties of these derivatives and pave the way for newer treatment strategies.

## 6. Metabolism of Resveratrol

Various experimental strategies have been employed to study and demonstrate the metabolism of resveratrol. It has been observed that the phase II metabolic enzymes as well as the intestinal microorganisms are crucial for its biotransformation. Additionally, its rate of metabolism also depends on the dose administered, present disease states, sex and tissues among others [73].

The liver is responsible for the glucuronidation of resveratrol, along with the intestinal microbiota. Its glucuronidation leads to the formation of two conjugates, namely, resveratrol 3-O-glucuronide and resveratrol 4′-O-glucuronide. UGT1A1 and UGT1A9 are primarily involved in the development of the 3-O-glucuronide (Km = 149 microM) and 4′-O-glucuronide (Km = 365 microM), respectively. The glucuronide conjugates are generated at a greater extent (up to 10-times) by the intestinal microsomes, in contrast to the liver microsomes [74].

The UGT family of enzymes are involved in the catalysis reaction of conjugation of resveratrol with a glucuronic acid moiety either at the 3 or 4′ hydroxyl group position, thereby changing the pharmacological properties of the antioxidant compound and promoting its elimination from the body. The human liver microsomes (HLMs) contain abundant UGT enzymes, and selectively form greater amounts of 3-O-glucuronide, as compared to 4′-O-glucuronide [75].

Sulfation is another mechanism of metabolism of resveratrol. A study showed that human sulfotransferase (SULT) 1A1, 1A2, 1A3, and 1E1 led to the sulfation of resveratrol to different degrees, forming up to three metabolites, resveratrol-3-O-sulfate, resveratrol-4′-O-sulfate, and resveratrol-3, 4′ -O-disulfate. These are the primary sulphated conjugates of resveratrol [76]. Resveratrol-3-O-sulfate is nearly selectively produced by SULT1A2 and 1A; as compared to SULT1A2 and 1A3, SULT1E1 demonstrates lower catalytic capability to produce resveratrol-3-O-sulfate [77].

Resveratrol exhibits antioxidant and anti-inflammatory properties, thus making it a potential anti-cancer agent. It inhibits the production of pro-inflammatory factors by causing the SIRT1 gene activation. It causes the inhibition of RelA acetylation which reduces NF-κB-induced expression of inflammatory mediators including Interleukins (namely, IL-1β, IL-10 and IL-6), TNF-α, Cox-2, metalloproteases (MMP)-1 as well as MMP3 [78].

The cell-selective effect on the formation of interleukin is an important characteristic of resveratrol. Resveratrol is seen to increase the production of IL-1β and IL-6 in the peripheral blood lymphocytes (PBLs); however, it exhibits the opposite effect in macrophages [79].

It causes the transformation of growth factor-beta (TGF β), as well as the inhibition of inflammatory pathways mediated by Toll-like receptors [80]. Studies have also shown that resveratrol lowers the activity of TANK-binding kinase1 (TBK1) as well as receptor-interacting protein 1 (RIP1) in a toll-interleukin-1 receptor domain comprising an adaptor activating an interferon (TRIF) complex within the myeloid differentiation factor 88 (MyD88)-independent signaling pathways [81].

In addition, studies have confirmed that resveratrol administration can be a potential treatment strategy against lupus nephritis in MRL/lpr mice by enhancing the activity of FcγRIIB, enabling the selective decrease of B cells in the spleen as well as bone marrow [82].

Resveratrol, at a low dose, inhibits the production and activity of tumor evoked regulatory B-cells by causing the inhibition of Stat3. This leads to the inhibition of the expression of TGFβ, a downstream target of Stat3 [83].

There is numerous experimental data describing the various regulatory mechanisms and the immunomodulatory activity of resveratrol both under in vivo and in vitro settings. Further clinical trials are awaited to enhance the bioavailability of the compound and understand its mechanism of action in physiological conditions of malignancies [84].

**Figure 2:**
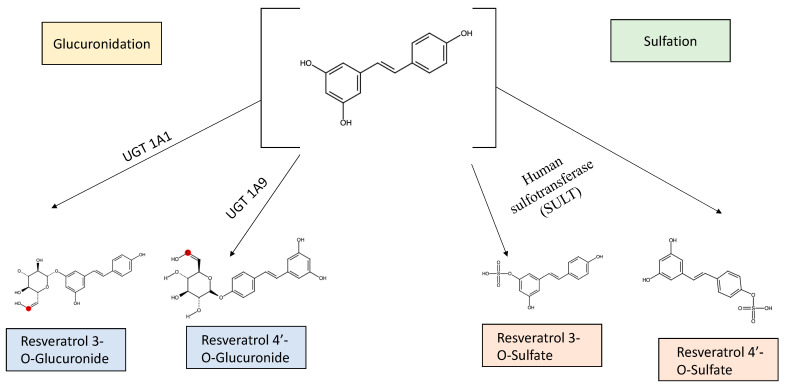
Representation of the metabolism of Resveratrol along with its main metabolites.

Figure 2 offers a representation of the metabolism of resveratrol, along with its main metabolites.

## 7. Nanotechnological Interventions

A major challenge with the delivery of Resveratrol has been low bioavailability, owing to its poor solubility (reported to be lower than 0.05 mg/mL) [10]. A study undertaken by Li et al. explored the solubility of resveratrol in peanut oil, and up to 95% solubility was achieved on optimization of mixing conditions. Even in a lipid-based solvent, resveratrol was able to retain its antioxidant property, along with an improvement of its shelf life. This indicated potential for using peanut oil as a carrier for the drug, during formulation designing [85].

However, a need has been felt to improve the solubility and stability of this phytochemical, and various nanotechnological interventions have been employed. Various nanoformulations have been employed to improve the residence time of the drug in the body, as well as subsequent improvement of bioavailability [86]. In addition to these benefits, another major advantage of nanoformulation usage is the reduced metabolism of the drug, chiefly by the inhibition of glucuronidation [87]. Therefore, nanotechnological interventions present a tremendous opportunity to improve the delivery of this agent, and minimize limitations associated with its stability and solubility. Some such examples have been summarized in the table below (Table 2).

## 8. Marketed Formulations of Resveratrol

Resveratrol is a naturally occurring polyphenol, but it is being synthesized artificially for therapeutic use due to its low yield on isolation. It is currently marketed in various traditional dosage forms such as tablets and capsules. Table 3 gives an overview of the main marketed formulations of resveratrol, along with their indications and dosage.

## 9. Challenges and Future Perspectives

Resveratrol, a widely known polyphenolic stilbenoid, has been assessed for its application in the management of a variety of neoplasms, including B cell malignancies. Its wide availability and versatility have aided its applications, presenting it as a promising molecule for subsequent clinical usage.

With respect to epidemiological data obtained from the United States and Western Europe, projections indicated an increase in patients owing to a low rate of initiation of systemic treatments. In addition to this, rising incidence may be linked with a growing ageing population, toxicity resulting from accumulation of therapeutic agents over time, as well as a rising rate of relapse [97]. In addition to this, it is important to understand the genetic basis for disease incidence as well. A study conducted by Din et al. indicated similarities between genetic factors causing autoimmune disease and non-Hodgkin lymphoma. An overlap in risk factors may indicate the possibility of cancer occurrence if these genetic mutations are not controlled [98]. Another study undertaken by Chang et al. explored the relation between family history and a predisposition to hematopoietic malignancies. A significant risk of occurrence was observed if a first-degree relative had a history of cancer, and while a strong relation with environmental factors was not established, genetics play an important role in determining the occurrence of B cell malignancies [99]. Despite being prominent consumers of wine, these countries show a greater incidence of B cell malignancies, relative to the rest of the world. A prime reason for this may also be the low solubility and stability of resveratrol, thereby directly affecting its bioavailability, as discussed previously. Novel therapeutic approaches are being employed to overcome these challenges; however, the progress remains slow. The factors highlighted above may play an important role in this, and it has been understood that a variety of factors, in addition to dietary supplementation, must be analyzed in order to arrest disease progression and improve treatment outcomes.

Although a vast number of pre-clinical studies have been conducted to assess the pharmacological benefits of resveratrol, significant advances in clinical research are yet to be made. This stimulates the need for clinical trials in diverse populations, and a thorough assessment of the safety and toxicity profile of this agent, taking into consideration its interactions with other phytochemicals as well as synthetic anti-cancer compounds. Owing to its natural origin, it poses several formulation challenges, such as low solubility and stability, as well as rapid systemic metabolism, which reduces its delivery to target tissues and affect its bioavailability. This, in turn, hinders the achievement of desired therapeutic outcomes. In addition to this, nephrotoxicity resulting from resveratrol administration has been observed in patients with multiple myeloma [11], and there is a need to evaluate the risk of toxicity associated with long-term administration for chronic diseases. These challenges must be addressed before amplifying its exploration as an anti-cancer agent, as well as potential avenues for other applications.

As the focus on nanotechnological delivery continues to grow, in order to tackle challenges with respect to optimizing drug delivery, various nano-strategies have been adopted to enhance the bioavailability of this agent. Some of these therapeutic strategies have been highlighted by the authors, with the aim to spark discussion with respect to the applicability of these nanoformulations. However, a major gap that remains to be addressed is the low potency of existing formulations, which may be enhanced by undertaking synthesis of analogues of resveratrol, some of which have been discussed, to significantly improve its anti-cancer effects.

While resveratrol has been observed to be well tolerated over both short- and long-term dosing in healthy patients, it is essential to establish its safety in sick patients. There is a need to conduct a greater number of focused, clinical studies across various demographics, to develop a complete understanding of the safety and efficacy of this molecule, as well as its pharmacological potential.

## Figures and Tables

**Table 1:** Pre-clinical studies of resveratrol in the management of B-cell malignancies.

Agents Used	Animal Model/Cell Line/Samples Used, and Study Type	Dose and Duration of the Study	Molecular Mechanisms	Conclusion	Reference
Resveratrol isolated from black grape skin (*Vitis vinifera*)	Blood samples from patients with Hodgkin lymphoma (HL) and non-Hodgkin lymphoma (NHL)	Lowest dose of 50 µg/mL, ranging up to 1000 µg/mL	Lowering of inflammatory markers, such as TNF-α and IL-10 at low doses, inverse relation on increasing of dosage	Potential for usage as an anti-cancer agent, in patients suffering from lymphomas	[47]
Resveratrol pre-treatment followed by Paracetamol (PCM) dosage	Male Wistar rat model, with 6 rats in the focus group (pre-treatment+ PCM therapy)	Resveratrol (30 mg/kg, i.p.), for a week, followed by Paracetamol (2 g/kg, orally)	Controlled expression of B-cell lymphoma 2 (Bcl-2) in the rat model	Reduction in inflammation, triggering of apoptosis, exertion of protection by resveratrol in a model of acute liver injury, indicating applications in a variety of conditions involving impaired Bcl-2 expression, such as lymphomas	[48]
Resveratrol in varying concentrations	Treatment of MOLT-4 and HL-60 cell lines	For MOLT-4 cell line: 41 μM, up to 48 h; for HL-60 cell line: 43 μM, up to 72 h	Reduction of cellular viability, modulation of cell cycle as well as levels of key proteins associated with autophagy, triggering of apoptosis by caspase-3 activation	Resveratrol may be employed to control tumor growth, by triggering apoptosis and autophagy. It demonstrates a satisfactory safety and solubility profile, indicating wider usage for leukemia management, following a greater number of clinical studies	[49]
Resveratrol+ Prednisolone	CCRF-CEM cells (human leukemia cell line)	Varying doses of 15, 50, 100 μM of resveratrol, followed by 700 μM of prednisolone	Reduced expression of multidrug protection quality 1 (MDR-1), thereby exerting cellular protection	Based on the outcome of the study, resveratrol was observed to exert a cytoprotective effect, as well as lowering the incidence of drug resistance brought about by MDR-1, indicating potential in lymphoblastic leukemia management	[50]
Resveratrol	Human normal peripheral blood PBMC cells, and human acute promyelocytic leukemia (APL) cell line, NB-4 and HL-60 cell lines	Varying concentrations of resveratrol (0, 5, 10 and 20 μM)	Improved activity of caspase-3, induction of apoptosis, lowered expression of p-AKT	By modulating the PI3K/AKT pathway, resveratrol exerted an anti-cancer action, indicating a scope for anti-leukemic applications in clinical settings	[51]
Methoxy-analogues of resveratrol	Human promyelocytic (HL-60) and monocytic leukemia (THP-1) cell lines	Varying concentrations of resveratrol (1–200 μM), treatment for 24 h	Greater cytotoxic potential of the analogues as compared to unmodified resveratrol, greater rate of apoptosis through upregulation of proteins such as Bax	Methoxy derivatives of resveratrol may be employed to bring about cell cycle arrest and apoptosis in leukemic cells, showing a promising potential for application	[52]
Resveratrol, in combination with prednisolone	CCRF-CEM cell line	Varying concentrations of resveratrol (15, 50 and 100 µM), along with prednisolone (700 µM), analysis up to 48 h following treatment	Improvement in the action of glucocorticoids such as prednisolone, induction of apoptosis by upregulation of proapoptotic proteins and downregulation of proteins like Bcl2, responsible for inhibition of apoptosis	Resveratrol may be employed in the management of leukemic conditions, in conjunction with synthetic agents, in order to improve therapeutic outcomes	[53]
Resveratrol	K562/ADM cell line	Concentrations ranging from 0–80 μmol/L, treated up to 72 h	Induction of cellular autophagy, reduced expression of Bcl-2, increased activity of caspase-3	Resveratrol was observed to reduce the viability of cancer cells, through the triggering of an apoptotic cascade. It exerted a cytoprotective action on healthy cells, thereby indicating potential for usage in the management of leukemia	[54]

Legend: HL—Hodgkin lymphoma, NHL- Non-Hodgkin lymphoma, TNF-α, IL-10, PCM—Paracetamol, Bcl-2—B-cell lymphoma 2, MDR-1: Multidrug protection quality 1, APL—Acute promyelocytic leukemia, p-AKT: Protein kinase B.

**Table 2:** Nanotechnological advancements in resveratrol delivery.

Nanoformulations	Chemical Composition	Key Benefits Offered	Reference
Resveratrol loaded nanoparticles (NPs)	Chitosan and γ-poly(glutamic acid) (γ-PGA)	Improved UV stability, enhanced solubility and antioxidant property	[88]
Nanocomplexation of resveratrol with nanofibrils	Fabricated pea protein isolate (PPI) nanofibrils+ resveratrol	Significantly improved solubility, greater surface area for drug incorporation, greater antioxidant potential even at low doses	[89]
Resveratrol loaded onto nanosponges	Combination of resveratrol and oxyresveratrol	Improved UV stability, solubility as well as antioxidant effect, as well as a satisfactory toxicity profile	[90]
Resveratrol nanofibers	Loading of the drug onto polyvinylpyrrolidone/cyclodextrin nanofibers, prepared by electrospinning	Better solubility profile, satisfactory extent of penetration as well as improved antioxidant activity	[91]
Resveratrol nanoparticles	Loading of the drug onto a chitosan-pectin core	Provision of sustained drug delivery, improved activity, easy modulation of release by variation of parameters	[92]
Self-emulsifying drug delivery system (SEDDS) of resveratrol	Usage of cod liver oil, as well as surfactant systems such as sodium oleate, Tween 80, alongside colloidal carriers	Decreased cohesive force, resulting in improved oral bioavailability	[93]
Resveratrol Microparticles	Usage of magnesium dihydorixde as a supporting base	Improvement of solubility and subsequently bioavailability	[94]
Resveratrol + Gefitinib cocrystals	Combination of resveratrol with a synthetic chemotherapeutic agent	Improved stability as well as solubility, indicating potential for increased clinical usage	[95]
Resveratrol encapsulated with silica carriers	Encapsulation of the drug alongside functional silica carriers, matrix-type drug release	Maintenance of cytotoxic properties, improvement of solubility profile	[96]

Legend: NPs: Nanoparticles; γ-PGA: γ-poly(glutamic acid); PPI: Pea protein isolate, SEDDS: Self-emulsifying drug delivery system.

**Table 3:** Overview of the marketed formulations of resveratrol, along with their indications and dosage.

Composition	Product	Dose	Indication and Key benefits
Resveratrol red wine extract capsules	21st century resveratrol red wine extract- Dietary supplement	200 mg red wine complex+ Ascorbic acid (60 mg), Once a day	Reduction of oxidative stress, improvement of immune system functioning, reduction in inflammation
Trans-Resveratrol, sourced from *Polygonum cuspidatum* root	Doctor’s best High potency Trans-resveratrol capsules	600 mg resveratrol, one vegetable gelatin capsules a day	Lowered oxidative damage, protection of cells from reactive oxygen species (ROS) and free-radical damage, potential to cross the blood-brain barrier (BBB)
Resveratrol, in addition to Omega-3 fatty acids, Zinc, Chromium and Selenium	Resvita Capsules, Aristo pharmaceuticals Pvt Ltd.	5 mg resveratrol, one capsule a day	Antioxidant potential, reduction of inflammation, triggering of cellular apoptosis
Resveratrol+ Nicotinamide mononucleotide (NMN)	Lifespan supplements- Nutraceutical capsules	300 mg resveratrol, once a day	Improved vascular functioning, enhancement of DNA repair, elevated mitochondrial functioning, improvement in NAD+ levels
Trans-resveratrol+ Ascorbic acid (Vitamin C)	Youtheory resveratrol tablets	250 mg resveratrol, up to 4 tablets per day	Improved bioavailability, protection against oxidative stress, neutralization of free radicals
Trans resveratrol (sourced from *Polygonum cuspidatum* root)+ *Piper nigrum* extract	Trans resveratrol-500 mg-30 Vcaps^®^ Plus- Aarogya 360+	500 mg Trans-resveratrol, 1 capsule twice a day	Mitochondrial biogenesis, antioxidant properties, cardio and neuroprotective effects
Resveratrol (sourced from *Polygonum cuspidatum* root)	Extra strength resveratrol capsules, NOW foods	350 mg resveratrol	Improved response to biological stress, free radical scavenging, supporting of cellular health
Resveratrol	Resveratrol Defense Quick release capsules-Piping Rock (n = 180)	100 mg resveratrol, 1 capsule up to 4 times daily	Improved release profile, improved cellular functioning, reduction in oxidative stress, reduced inflammation
Bioactivated Resveratrol- Red grape extract	Red grape antioxidant bioactivated resveratrol capsules- Nature’s goodness (n = 60)	500 mg resveratrol, 1 capsule a day	Lowered oxidative stress, enhancement of cellular functioning, lowering of inflammatory markers
Resveratrol	Resveratrol Complex Capsules- Swanson (n = 60)	100 mg resveratrol, 1 capsule a day	Protection from free radicals, cellular longevity, promotion of overall health

Legend: ROS: Reactive oxygen species, BBB: Blood brain barrier, NMN: Nicotinamide mononucleotide.

## Data Availability

Data sharing is not applicable to this article as no datasets were generated or analyzed during the current study.
